# Commentary on “The use of laser in urogynaecology”

**DOI:** 10.1007/s00192-019-03885-1

**Published:** 2019-02-02

**Authors:** Adolf Lukanović

**Affiliations:** Department of Gynecology, University Medical Centre Ljubljana, Šlajmerjeva 3, SI-1000 Ljubljana, Slovenia

Dear Editor,

We read with interest the recent review “*The use of laser in urogynaecology*” by Bhide and coauthors [1]. Since the use of laser in urogynaecology has been increasing in recent years, there is a great need for quality systematic reviews to provide evidence-based guidance to health care providers. Unfortunately, Bhide et al. failed to include several relevant studies [2, 3] in their review and we fear this may have affected their conclusions. The “Methods” section of the review has insufficient detail on the database search and inclusion and exclusion criteria for the selected literature [1], which weakens the scientific rigour of the review.

Bhide et al. cited a recent review by Gambacciani and Palacios [4], but omitted several studies included therein without explanation. A notable example is a study by Tien et al. published in 2016 in International Urogynecology Journal [2]. Thirty-five women with urodynamic stress urinary incontinence (SUI) were included in this prospective study and received a single treatment with a non-ablative vaginal erbium laser. In addition to a comprehensive collection of questionnaires and bladder diary, the study also included urodynamic tests and 20-min pad tests, objective outcome measures missing from many studies included in Bhide et al. A significant reduction of average pad weight from 14.0 to 3.1 g as well as significant improvement in quality of life and sexual function was observed 6 months after treatment [2].

Bhide et al. conclude that there is a lack of randomised placebo-controlled trials concerning the effect of vaginal laser treatment for SUI but they missed the first one published in May 2018 [3]. In this randomised controlled trial including 114 women with SUI the improvement in ICIQ-UI SF, PISQ-12 and FSFI scores 3 months after single treatment was significantly better in the vaginal erbium laser group than in the sham control group [3].

A search for “stress urinary incontinence” and “laser” on the Clinicaltrials.gov database offers a great overview of the concluded studies as well many ongoing ones, including multi-centric randomised sham-controlled trials, which might alleviate some of the authors’ pessimism regarding the present and future quality of evidence on the topic. We highly recommend this database to all researchers to assure their reviews are timely and up to date.
